# Exploring the nexus between organizational silence and hospital magnetism: A cross-sectional study on clinical nurses in China

**DOI:** 10.1097/MD.0000000000049928

**Published:** 2026-07-31

**Authors:** Hui-Ying Chen, Li-Hua Shen, Si-Jia Li, Jia Guo, Wen Zhang, Chen-Ping Zhu, Juan Tao

**Affiliations:** a Nursing Department, Sir Run Run Shaw Hospital, Zhejiang University School of Medicine, Hangzhou, Zhejiang, China.

**Keywords:** hospital magnetism level, Magnet hospital, nurses, organizational silence

## Abstract

Organizational silence, referring to employees’ reluctance to voice their opinions and constructive suggestions, can have an adverse impact on hospitals, individuals, and patient safety. Magnet hospitals, which are renowned for attracting and retaining nursing talent, cultivate a more supportive work environment. However, there is a significant knowledge gap regarding the relationship between hospital magnetism levels and organizational silence among clinical nurses in China’s Magnet hospitals. This study fills that gap. In March 2024, a cross-sectional survey at a Magnet-certified Grade A hospital in Zhejiang enrolled 390 clinical nurses via convenience sampling. Data were collected electronically using the Employee Silence Behavior Survey Scale and the Chinese Version of the Essentials of Magnetism Scale II. Overall and item - level mean scores for organizational silence and hospital magnetism were calculated. Pearson correlation coefficients were used to examine the relationship between organizational silence and hospital magnetism. Multiple linear regression analysis was performed, with organizational silence as the dependent variable and hospital magnetism as 1 independent variable, along with other significant demographic and occupational characteristics, to identify predictive factors. A total of 390 clinical nurses were included (89.7% under 40 years old, 98.5% female). The mean organizational silence score was 31.32 ± 11.02, and the mean hospital magnetism score was 152.88 ± 21.96. Hospital magnetism was negatively correlated with organizational silence (*r* = −0.488, *P* < .01). Multiple linear regression showed that unmarried status (β = 0.18, *P* < .01), lower professional title (β = −0.30, *P* < .01), poorer self-perceived health (β = 0.12, *P* = .01), and lower hospital magnetism (β = −0.44, *P* < .01) were significant predictors of higher organizational silence. The model explained 28% of the total variance in organizational silence, and hospital magnetism alone explained 17.7%. This is the 1st study to investigate the relationship between organizational silence and hospital magnetism levels among clinical nurses in China’s Magnet hospitals. The findings confirm a significant negative correlation between the 2, with hospital magnetism emerging as a key influencing factor. We recommend that hospital managers focus on enhancing the magnetic work environment to reduce organizational silence.

## 1. Introduction

Organizational silence refers to the phenomenon in which employees, based on their knowledge and experience, can put forward constructive suggestions and ideas to improve certain aspects of their department or organization’s work, yet they hold back or refrain from expressing their personal opinions.^[1]^ Studies have indicated that organizational silence among nurses can have detrimental effects on the organization, individuals, and patient safety. Nurses tend to remain silent primarily because of “administrative and organizational reasons.”^[2,3]^ When nurses experience unfair treatment or feel excluded or ignored, they may choose to be silent, and this silent behavior is often positively correlated with the intention to leave.^[4]^ Moreover, when nurses lack a sense of support or psychological safety in the work environment, they are prone to remaining silent.^[5]^ Therefore, creating a positive and supportive work environment may be an effective strategy to reduce organizational silence among nurses.^[6]^

The concept of Magnet hospitals, 1st proposed by McClure and colleagues in the early 1980s, refers to hospitals that, even when facing a severe nurse shortage, can attract and retain nursing talent like a “magnet,” offering high-quality nursing services to patients.^[7]^ Magnet hospital accreditation is an assessment process carried out by the American Nurses Credentialing Center (ANCC) to determine whether the nursing services and the environment of a healthcare institution have reached an excellent level. A Magnet hospital must possess the 5 dimensions of the magnetic model, namely transformational leadership, structural empowerment, exemplary nursing professional practice, new knowledge, innovation and improvement, and empirical results. The accreditation is conducted every 4 years. Research has shown that Magnet hospitals offer a better nursing work environment and yield more positive outcomes for nurses, patients, and organizations compared to non-Magnet hospitals.^[8]^ By assessing nurses’ perception of hospital magnetism, the quality and health of a hospital’s work environment can be effectively reflected.^[9]^ However, little is known about the relationship between hospital magnetism and organizational silence among nurses, particularly in the Chinese context.

This research gap is particularly salient given the challenges faced by the nursing workforce in China. According to the National Health Commission (2023), China had 5.637 million registered nurses – only 4.00 per thousand population – which is below the World Health Organization’s recommendation of at least 5 nurses per thousand population. To compound this nursing shortage, the turnover rate of nurses in some regions of China was approximately 17.50%, while the proportion of nurses who reported an intention to leave was as high as 56.94%.^[10]^ High turnover rates exacerbate workload burdens, diminish job satisfaction, and threaten the quality of care. Organizational silence may be an important, yet under-studied, factor influencing nurses’ turnover intentions. As the 1st hospital in China to achieve Magnet designation (March 2019), Sir Run Run Shaw Hospital, Zhejiang University School of Medicine, provides a unique and valuable research setting to examine these issues. This study therefore aimed to examine the status of organizational silence among clinical nurses in a China’s Magnet hospital and explore its relationship with hospital magnetism levels. The findings are expected to provide evidence to support managerial strategies for reducing organizational silence and enhancing nursing retention and improving the quality of nursing practice environments.

## 2. Methods

### 2.1. Study design, population and sample size

This is a descriptive and cross-sectional study. Using convenience sampling, 390 clinical nurses were recruited from a Grade A comprehensive hospital in Zhejiang Province, China, which holds ANCC Magnet certification. These nurses were from departments such as internal medicine, surgery, intensive care, and the operating room, covering the vast majority of nursing departments in the hospital. Grade A hospitals are the highest-level medical institutions in China’s 3-tier hospital classification system and provide comprehensive healthcare services, clinical research, and medical education. The inclusion criteria were as follows: registered clinical nurses in active employment with a valid nursing practice certificate; willing to participate in this study. The exclusion criteria were as follows: nurses interning or studying at the hospital during the survey period; nurses on sick leave, personal leave, or maternity leave. According to Kendall method for estimating sample size in cross-sectional studies, the required sample size (N) should be 10 to 15 times the number of independent variables. In this study, a total of 11 independent variables were included. Based on this guideline, the minimum required sample size was estimated to be 11 × 10–11 × 15 = 110–165 participants. Considering a potential attrition rate of 20%, the final required sample size ranged from 110 × (1 + 20%)–165 × (1 + 20%) = 132–198 participants. Ultimately, 390 participants were enrolled, exceeding the minimum requirement.

### 2.2. Survey tools

#### 2.2.1. General demographic and occupational data

Based on a comprehensive literature review, the researchers developed a self-designed questionnaire, which collects information on gender, age, marital status, education level, years of clinical work experience, title, position (administrative roles), employment type, average monthly income, and self-perceived health status.

#### 2.2.2. Employee Silence Behavior Survey Scale (ESS)

The ESS was developed by Zhen et al in 2008 to assess the level of organizational silence among employees.^[1]^ The scale includes acquiescent silence (items 1–4), defensive silence (items 5–8), and indifferent silence (items 9–12). Acquiescent silence refers to the passive compliance of employees who are unable to change the current situation. Defensive silence mainly describes the self-protection of employees to avoid interpersonal estrangement and attacks from others. Indifferent silence mainly describes the passive retention of opinions by employees due to their insufficient attachment and identification with the current job or organization. Using a 5-point Likert scoring system, from “strongly disagree” to “strongly agree” scored 1 to 5 points, with a total score ranging from 12 to 60 points. The total score for each dimension is 4 to 20 points. Higher scores indicate a higher level of organizational silence. Initially, this scale was mainly used in management studies. In recent years, it has also been widely used by nursing scholars in related surveys of organizational silence among nurses, with Cronbach α coefficient ranging from 0.79 to 0.92, proving its applicability to the nursing population.^[11]^ In this study, the Cronbach α coefficients for the total ESS and its 3 dimensions were 0.91, 0.87, 0.85, and 0.88, respectively, indicating excellent internal consistency. (Supplemental Digital Content, Supplemental Digital Content 1).

#### 2.2.3. Chinese Version of the Essentials of Magnetism Scale Ⅱ (EOM Ⅱ)

The EOM Ⅱ was revised by Pan et al based on the hospital magnetism scale compiled by Kramer et al, which is used for the evaluation of the hospital nursing work environment and magnetism level.^[9,11]^ The scale includes 7 dimensions: nursing management support, cultural values, autonomy in nursingwork, –physician–nurse relationships, management of nursing practice, educational support, and reasonable allocation of nursing human resources, with a total of 45 items. Each item uses a Likert 4-point scoring method, from “strongly disagree” to “strongly agree,” scored from 1 to 4 points. The total score ranges from 45 to 180 points. A higher score indicates a healthier nursing work environment and a higher magnetism level. The total scale Cronbach α coefficient of this scale is 0.90, and the Cronbach α coefficients for each dimension range from 0.84 to 0.93. In our sample, the Cronbach α for the total EOM Ⅱ was 0.93, with dimension-specific α ranging from 0.86 to 0.91, confirming high reliability. (Supplemental Digital Content, Supplemental Digital Content 1).

### 2.3. Data collection method

Data collection was conducted online via the Questionnaire Star platform over a 2-week period in March 2024. The average time to complete the questionnaire was approximately 10 to 15 minutes. A pilot test was conducted with 30 nurses to ensure the clarity and feasibility of the questionnaire. Minor wording adjustments were made based on the feedback, and data from the pilot study were not included in the final analysis. Through the hospital work communication group, eligible participants were recruited, and electronic questionnaires were distributed to voluntarily participating nurses, inviting them to fill them out. The research team developed the questionnaires using the Questionnaire Star platform, and all questions were set as mandatory. An IP address restriction was applied to ensure that the questionnaire could be filled out only once to avoid repeated submissions. Prior to completing the questionnaire, participants were informed of the purpose and significance of the study, the method of answering, obtained the informed consent of the research subjects, and promised to keep all the filled information confidential.

### 2.4. Statistical analysis

The data were analyzed using SPSS version 26.0. Categorical data were described using frequencies and percentages, while continuous data were described by the mean ± standard deviation. The comparison between groups was conducted using a *t* test. Pearson correlation analysis was employed in the correlation study.

To identify factors influencing organizational silence, a multiple linear regression analysis was performed. In this analysis, the total score of organizational silence was taken as the dependent variable, and the hospital magnetism level and the variables with statistically significant differences in univariate analysis were used as independent variables. A –*P* value <.05 was considered statistically significant.

## 3. Results

### 3.1. Participant characteristics

A total of 390 clinical nurses participated in this study. The majority were female (384, 98.5%), and 89.7% were under 40 years old. Nearly half were unmarried (48.5%), while 51.5% were married. Most nurses held a bachelor’s degree (93.6%), and 71.5% had 10 years or less of clinical work experience. Contract workers accounted for 59% of the sample, and 78.5% reported a monthly income between 5000 and 20,000 yuan. Regarding departmental distribution, 112 nurses (28.7%) worked in internal medicine, 98 (25.1%) in surgery, 85 (21.8%) in intensive care units, 65 (16.7%) in the operating room, and 30 (7.7%) in other departments.

### 3.2. Organizational silence

Table 1 presents the level of organizational silence. The overall level was moderate (mean item score = 2.61 ± 0.92). Among the 3 dimensions, acquiescent silence was the most prevalent, followed by defensive silence, while indifferent silence was the least common.

**Table 1:** Scores of organizational silence among clinical nurses in a Magnet-certified hospital in Zhejiang Province, China (March 2024, n = 390).

Dimensions	Number of items	Overall dimension score (mean ± SD)	Item average score (mean ± SD)
Acquiescent silence	4	11.60 ± 4.38	2.90 ± 1.09
Defensive silence	4	10.70 ± 4.05	2.67 ± 1.01
Indifferent silence	4	9.03 ± 3.81	2.26 ± 0.95
Total ESS	12	31.32 ± 11.02	2.61 ± 0.92

ESS: Employee Silence Behavior Survey Scale, with higher scores indicating more severe silent behaviour.

ESS = Employee Silence Behavior Survey Scale, SD = standard deviation.

### 3.3. Hospital magnetism level

Table 2 presents the levels of hospital magnetism across its 7 dimensions. Overall, nurses reported a generally positive perception of hospital magnetism (mean item score = 3.40 ± 0.49). “Cultural Values” scored the highest, whereas “Nurse–Physician Relationship” received the lowest score.

**Table 2:** Scores of clinical nurses’ perception of hospital magnetic level in different dimensions in a Magnet-certified hospital in Zhejiang Province, China (March 2024, n = 390).

Dimensions	Number of items	Overall dimension score (mean ± SD)	Item mean score (mean ± SD)
Cultural values	10	34.96 ± 4.98	3.50 ± 0.50
Educational support	4	13.69 ± 2.18	3.42 ± 0.54
Support from nursing management	13	44.49 ± 7.12	3.42 ± 0.55
Autonomy in nursing work	7	23.52 ± 3.46	3.36 ± 0.49
Management of nursing practice	4	13.37 ± 2.29	3.34 ± 0.57
Rational allocation of nursing human resources	3	9.93 ± 1.80	3.31 ± 0.60
Nurse-physician relationship	4	12.92 ± 2.38	3.23 ± 0.59
Total EOM Ⅱ	45	152.88 ± 21.96	3.40 ± 0.49

Dimensions are ordered by item mean score from highest to lowest.

EOM II = Chinese Version of the Essentials of Magnetism Scale Ⅱ, SD = standard deviation.

### 3.4. Univariate analysis of factors influencing organizational silence

Table 3 shows that organizational silence varied significantly by nurse characteristics. Higher silence levels were observed among unmarried nurses, those with ≤5 years of work experience, those with junior titles or below, and those with lower income or poorer health. Head nurses reported significantly lower silence levels than staff nurses.

**Table 3:** Comparison of organizational silence and hospital magnetism scores of clinical nurses with different characteristics (n = 390).

Items	Number of nurses	Organizational silence score (mean ± SD)	*t*/*F* value	*P* value	Magnetism level score (mean ± SD)	*t*/*F* value	*P* value
Gender							
Male	6	40.17 ± 21.79	1.01	.36	172.17 ± 18.23	2.18	.03
Female	384	31.18 ± 10.76			152.58 ± 21.90		
Age							
≤29	189	33.60 ± 10.76	2.13	.10	150.00 ± 21.54	2.13	.10
30–39	161	28.56 ± 10.54			155.52 ± 20.95		
40–49	38	31.84 ± 11.85			155.82 ± 26.56		
≥50	2	28.50 ± 16.36			157.00 ± 32.53		
Marital status							
Unmarried	189	32.53 ± 10.36	2.52	.01	149.90 ± 21.52	−2.53	.01
Married	201	29.75 ± 11.21			155.53 ± 21.91		
Education level							
Bachelor	365	31.37 ± 10.93	0.32	.75	152.76 ± 22.13	−0.41	.68
Postgraduate	25	30.64 ± 12.48			154.64 ± 19.68		
Clinical working years							
≤5	175	33.68 ± 10.65	6.44	<.01	149.01 ± 21.52	4.68	<.01
6–10	104	28.50 ± 10.45			156.23 ± 20.80		
11–20	88	31.13 ± 11.66			153.80 ± 23.67		
>20	23	26.91 ± 10.00			163.74 ± 18.00		
Title							
Nurse	91	34.03 ± 11.04	7.97	<.01	149.49 ± 22.12	3.76	<.01
Senior nurse	102	33.81 ± 10.62			149.20 ± 20.95		
Supervisor nurse	189	28.98 ± 10.68			156.02 ± 22.10		
Chief nurse	8	24.13 ± 9.64			164.25 ± 17.46		
Position							
Staff nurse	373	31.72 ± 10.98	3.36	.01	152.33 ± 22.07	−3.20	<.01
Head nurse	17	22.65 ± 8.05			164.94 ± 15.55		
Employment type							
Regular staff	160	30.29 ± 11.48	−1.55	.12	154.17 ± 22.29	0.97	.34
Contract worker	230	32.04 ± 10.65			151.99 ± 21.73		
Monthly income (CNY)							
≤5000	74	35.03 ± 10.34	4.42	<.01	148.78 ± 24.60	2.30	.09
5001–10,000	157	31.27 ± 11.63			151.72 ± 21.94		
10,001–20,000	149	29.86 ± 10.49			155.64 ± 20.57		
>20,000	10	26.50 ± 11.02			160.40 ± 16.80		
Self-perceived health status							
Healthy	196	29.18 ± 11.07	9.86	<.01	157.81 ± 20.18	12.21	<.01
Average	186	33.16 ± 10.31			148.50 ± 21.44		
Poor	8	41.13 ± 14.17			134.13 ± 21.96		

### 3.5. Univariate analysis of factors influencing hospital magnetism level

Table 3 shows that higher hospital magnetism levels were significantly associated with being male, married, having >20 years of experience, holding mid-level or higher titles, serving as a head nurse, having higher income, and better self-perceived health.

### 3.6. Correlation analysis of organizational silence and hospital magnetism level

Table 4 shows that the overall correlation coefficient between organizational silence and hospital magnetism was −0.488 (*P* < .01), indicating a moderate-to-strong negative correlation. Among the dimensions, “Support from nursing management” showed the strongest negative correlation with organizational silence, followed by “Cultural values.”

**Table 4:** Correlation analysis (Pearson *r*) between organizational silence and magnetic level in clinical nurses.

Items	Organizational silence	Acquiescent silence	Defensive silence	Indifferentsilence
Magnetism level	−0.488	−0.468	−0.409	−0.439
Nurse-physician relationship	−0.361	−0.351	−0.280	−0.342
Educational support	−0.464	−0.443	−0.381	−0.428
Autonomy in nursing work	−0.404	−0.359	−0.353	−0.383
Management of nursing practice	−0.438	−0.424	−0.371	−0.387
Rational allocation of nursing human resources	−0.440	−0.435	−0.359	−0.389
Support from nursing management	−0.475	−0.488	−0.384	−0.407
Cultural values	−0.497	−0.396	−0.403	−0.411

All *P*<.01. Correlation coefficient (*r*) interpretation: ±0.1 to ± 0.3 = weak; ±0.3 to ± 0.5 = moderate; >±0.5 = strong.

### 3.7. Multivariate analysis of organizational silence

Tables 5 and 6 show that multivariate analysis identified marital status, professional title, self-perceived health, and hospital magnetism level as significant predictors of organizational silence, collectively explaining 28% of the variance. Hospital magnetism alone accounted for 17.7% of the variance, with higher magnetism predicting substantially lower silence scores.

**Table 5:** Assignment methods of argument variables.

Independent variables	Assignment method
Marital status	Unmarried = 1, married = 2
Clinical working years	≤5 yr = 1, 6–10 yr = 2, 11–20 yr = 3, >20 yr = 4
Title	Nurse = 1, senior nurse = 2, supervisor nurse = 3, chief nurse = 4
Position	None = 1, head nurse = 2
Monthly income	≤5K income = 1, 5–10K income = 2, 10–20K income = 3, >20K income = 4
Self-perceived health status	Healthy = 1, average = 2, poor = 3
Magnetism level	Original value

Marital Status: “Married” as reference; working years: “≤5 years” as reference; title: “Nurse” as reference; position: “None” as reference; Monthly income:“≤5000 income” as reference; self-perceived health status: “Healthy” as reference.

**Table 6:** Multivariate linear regression analysis of influencing factors of clinical nurses’ organizational silence.

Variables	Partial regression coefficient	Standard error	Standardized regression coefficient	*T* value	*P* value	95% CI
Constant	66.83	4.77		14.00	<.01	57.45–76.21
Marital status	3.60	1.26	0.18	2.87	<.01	1.13–6.07
Clinical working years	0.43	0.85	0.04	0.50	0.62	−1.25 to 2.11
Title	−3.86	1.12	−0.30	−3.46	<.01	−6.06 to −1.67
Position	−2.99	2.52	−0.06	−1.19	.24	−7.94 to 1.96
Monthly income	0.07	0.85	0.01	0.08	.94	−1.61 to 1.74
Self-perceived health status	2.38	0.92	0.12	2.59	.01	0.57–4.18
Magnetism level	−0.22	0.02	−0.44	−9.75	<.01	−0.26 to −0.18

Marital status: “Married” as reference; working years: “≤5 years” as reference; title: “Nurse” as reference; position: “None” as reference; monthly income:“≤5000 income” as reference; self-perceived health status: “Healthy” as reference. *R*^2^ = 0.30, adjusted *R*^2^ = 0.28, *P*<.01.

## 4. Discussion

This study aims to explore the relationship between clinical nurses’ organizational silence and the magnetism level of the hospital in the context of Magnet hospitals. Through a survey of 390 clinical nurses from a tertiary grade A hospital in Zhejiang Province that holds ANCC Magnet certification, the following key results were obtained: the overall level of clinical nurses’ organizational silence was relatively low, with the score of acquiescent silence being the highest. The overall magnetism level of clinical nurses in the hospital is relatively high, with the score of the cultural values dimension being the highest and the score of the –doctor–nurse relationship dimension being the lowest. Marital status, professional title, and self-perceived health level are the influencing factors of clinical nurses’ organizational silence. Organizational silence is significantly negatively correlated with the magnetism level of the hospital, and the magnetism level can alone explain 17.7% of the total variation of organizational silence. The current study provides valuable insights into the relationship between organizational silence and hospital magnetism level among clinical nurses in China’s Magnet hospital.

### 4.1. The status of organizational silence among clinical nurses

In this study, the organizational silence behavior score of 390 clinical nurses was at a relatively low level (31.32 ± 11.02), lower than the survey results of non-Magnet hospitals (36.72 ± 12.56) in China. This indicates that the level of organizational silence among clinical nurses has improved to a certain extent in the context of Magnet hospitals.^[12–14]^ Reducing the level of organizational silence among nurses can effectively reduce the intention to leave, increase work engagement, and improve work performance levels.^[15]^ Among the 3 dimensions reported in this survey, the score of acquiescent silence was the highest. Acquiescent silence is the passive and negative withholding of opinions by employees who expect that they are unable to change the status quo, implying negative compliance.^[1]^ In contrast, the scores for defensive silence and indifferent silence were lower, indicating that nurses tended to have a weaker inclination to avoid being punished for their words or to show indifference towards the organization. Therefore, it is necessary to further cultivate a work atmosphere based on organizational trust, enhance nurses’ “sense of ownership,” increase employees’ recognition of the hospital, and encourage employees to think about and solve problems from the hospital’s common standpoint.

### 4.2. The status of hospital magnetism level among clinical nurses

The hospital magnetism level score of clinical nurses was at a relatively high level (152.88 ± 21.96, item mean 3.40), higher than the survey results of non-Magnet hospitals in China (132.28 ± 18.93) and also higher than the survey results in other countries.^[16–18]^ Evrenol^[19]^ reported that the EOM II item mean score was 2.2 (range 1.0–3.4) in Turkey. Our hospital was the 1st in China to carry out the Magnet hospital project by starting the preliminary work for the “Magnet Nursing Service Certification Project” in 2009,^[20]^ submitting the magnet certification application to the ANCC in 2012, and passing the Magnet hospital certification in March 2019.^[21]^ During the magnetic certification process, the hospital incorporated the core values of “honesty and sincerity, care and respect, cohesion and cooperation, integration and growth, and excellence and innovation” into its daily management. The results of this study show that the concept of Magnet hospital has been fully integrated into hospital management practices through the construction and certification process of Magnet hospitals, thereby enhancing clinical nurses’ perception of the magnetic environment. Among the dimensions, the cultural values dimension had the highest mean score, which may be due to the hospital’s emphasis on the construction of cultural values, making nurses highly recognize cultural values. The –nurse–physician relationship dimension had the lowest score. It is suggested that there is still room for improvement in the communication and collaboration between medical staff. Effective communication between medical staff is the foundation of hospital safety management.^[22]^ Therefore, hospital managers should pay more attention to maintaining –nurse–physician relationships.

### 4.3. Analysis of factors influencing organizational silence among clinical nurses

The research results also showed that clinical nurses who were unmarried, had shorter work experience, held junior titles, did not hold positions, had lower average monthly income, and had a poor self-perceived health status perceived a higher level of organizational silence. Specifically, unmarried nurses had significantly higher organizational silence scores. This may be because they tend to be younger, hold junior positions, and have fewer years of work experience and workplace seniority, resulting in lower confidence and more passive, acquiescent silence. The nurses with <5 years of work experience had the highest organizational silence scores, while those with >20 years of work experience had the lowest. This may be because senior nurses may have established closer working relationships with colleagues and superiors, which encourages them to communicate and share opinions more openly, thus reducing the occurrence of organizational silence.^[23]^ Similar conclusions have also been drawn in foreign studies.^[24,25]^ Junior nurses, being new to the workplace, often lack confidence in their professional skills and communication abilities, making them more prone to organizational silence. It is worth noting that the factors of professional title, position, and years of work are correlated. That is, junior nurses usually have lower professional titles, do not hold management roles, and have lower incomes. These factors collectively influence their organizational silence behavior. Nurses who feel their health is poor may lack the willingness to express themselves due to physical and mental exhaustion, or choose to remain silent out of fear that their health issues might affect their professional image. Therefore, hospitals should pay special attention to the psychological and professional adaptation of junior nurses. By implementing measures such as mentorship programs, communication training, and psychological support, they can foster a culture where nurses are willing to express themselves.

### 4.4. Analysis of factors influencing hospital magnetism level among clinical nurses

The research results showed that male, married clinical nurses with longer work experience, senior titles, positions, and good self-perceived health status perceived a higher hospital magnetism level. Although our subgroup analysis of male nurses (n = 6) suggested higher magnetism perception, the small sample size precludes robust conclusions. In China, male nurses are relatively scarce and are often assigned to high-demand departments such as the emergency department, intensive care unit, and operating room, with more prominent career development paths and management opportunities. Although there are no exclusive policies that only favor male nurses, their rarity and special role distribution may enhance their perceived organizational support and professional value, thus leading to higher magnetism scores. Future studies with larger and more balanced samples are needed to examine potential gender differences in the relationship between organizational silence and hospital magnetism. Married nurses reported higher magnetism perception, possibly because married nurses prioritize stable, supportive, and well-managed work environments. Magnet hospitals offer systematic career development, work-life support, and secure employment conditions that better meet the stability needs of married nurses. Monthly income was not significantly associated with magnetism perception, suggesting that magnetism reflects the organizational environment and professional support rather than monetary reward alone. Senior nurses with high seniority, senior titles, and positions have accumulated rich clinical practice experience over a long period, becoming the backbone of professional nursing fields, with more autonomy and say. Thus, they realize their professional value and have a higher perception of hospital magnetism.^[17]^ Junior nurses, having just entered the workplace, have limited understanding of the hospital’s culture and values, and their focus is more on how to be competent in clinical work. So, their perception of hospital magnetism may not be high. Open and supportive leaders play a crucial role in supporting nurses’ willingness to voice concerns, ideas, problems, or suggestions related to patient care.^[26]^ Therefore, hospitals should strive to create an open and inclusive work environment and establish an efficient feedback system to ensure that all nurses’ opinions are heard and taken seriously. Especially for junior clinical nurses with shorter work experience and lower titles, hospitals should pay special attention to help them adapt to the organizational culture more quickly and deepen their understanding and identification with the organization.

Nurses who feel in good health are energetic and highly engaged, and are more likely to perceive organizational support. Therefore, hospitals should enhance career development paths and health management to comprehensively increase nurses’ sense of identification with the magnetic environment.

### 4.5. The relationship between hospital magnetism level and organizational silence

Both correlation and regression analyses indicated that organizational silence was significantly negatively correlated with the hospital magnetism level (*r* = −0.488, *P* < .01), and the hospital magnetism level was an independent influencing factor for organizational silence, explaining 17.7% of the variance. This indicates that the higher the nurses’ perception of the hospital magnetism level, the lower the degree of organizational silence, which is consistent with previous research results.^[27]^

Administrative and organizational issues were often the main reasons for doctors and nurses in medical institutions to remain silent.^[28]^ The relationship between hospital magnetism and organizational silence can be theoretically grounded in organizational support theory. According to Eisenberger, 3 major categories of beneficial treatment perceived by employees – fairness, supervisor support, and organizational rewards and favorable job conditions – are positively associated with perceived organizational support.^[29]^ Within a magnetic hospital environment, each dimension of magnetism may foster organizational support through distinct mechanisms: leadership support directly encourages nurses to voice their opinions; autonomy reduces acquiescent silence by empowering nurses to act on their professional judgment; and positive –nurse–physician relationships lower defensive silence by minimizing interpersonal risk. It was reported that magnetic nursing workplaces can increase nurses’ professional recognition and reduce turnover.^[30]^ In a magnetic environment, clinical nurses can participate in department management through co-governance, empowering nurses with greater authority, fostering a sense of responsibility, and encouraging participation in joint decision-making, thereby stimulating nurses’ potential and giving them more chances to express themselves.^[31,32]^ Therefore, the level of organizational silence among clinical nurses will also be reduced in a work environment with a high level of magnetism. In addition, studies have shown that the sense of professional benefit among nurses can moderate the impact of part of the work pressure on organizational silence behavior, and the Magnet management model can reduce the work pressure of nurses and help nurses increase their sense of professional benefit.^[33]^ Nursing managers should continue to create a positive magnetic nursing environment, reduce the organizational silence behavior of nurses, promote communication and collaboration within the nursing team, and ultimately improve the quality of nursing, as well as patients’ outcomes and experiences.^[34,35]^

Therefore, we recommend 3 strategies to reduce organizational silence. While these strategies are also consistent with the Magnet model, they can be implemented independently, and each targets a specific type of silence. First, establish nurse-led shared governance committees at the unit and hospital levels to give nurses direct decision-making authority, thereby reducing acquiescent silence. Second, implement structured mentorship programs that pair junior nurses with senior preceptors to build confidence and communication skills, reducing defensive silence. Third, create anonymous, nonpunitive feedback channels to allow nurses to voice concerns without fear of reprisal, addressing indifferent silence.

## 5. Study strengths

This study is the 1st to explore the relationship between organizational silence and hospital magnetism among clinical nurses in a Chinese Magnet-certified hospital, filling the research gap in this field. The study used well-validated instruments with good reliability: the ESS and EOM II, which ensure the accuracy and credibility of the measurement results. The study hospital, through a decade-long systematic preparation and certification process, gradually integrated the magnetic concept into management practices, such as establishing a nurse shared governance committee and implementing an open communication mechanism. These experiences can provide a reference for other hospitals.

## 6. Limitations

While this study offers novel insights into the nexus between organizational silence and hospital magnetism among clinical nurses, several limitations must be acknowledged. Firstly, the cross-sectional design does not allow us to determine whether higher hospital magnetism reduces organizational silence or, alternatively, whether lower organizational silence enhances nurses’ perception of hospital magnetism. Future longitudinal or interventional studies are needed to establish temporal precedence and causal direction. Secondly, the study’s reliance on self-reported data through electronic questionnaires may introduce bias, as respondents might not accurately report their perceptions or behaviors. Social desirability bias could also influence the responses, particularly regarding sensitive topics such as organizational silence. Future studies could benefit from incorporating objective measures or 3rd-party validations to strengthen the validity of the findings. Thirdly, the sample, although representative of a Magnet-certified hospital in Zhejiang Province, may not be generalizable to all clinical nurses in China or other cultural contexts. The regional and hospital-specific characteristics could influence both organizational silence and hospital magnetism perceptions, suggesting a need for broader and more diverse samples in subsequent research. Additionally, while we controlled for several variables in our multivariate analysis, other potential confounders such as departmental culture, leadership styles, and specific work-related stressors were not accounted for. These factors could significantly influence organizational silence and hospital magnetism and merit inclusion in future studies. Lastly, the study’s focus on clinical nurses may overlook the perspectives of other hospital staff who also contribute to the organizational environment. Expanding the scope to include a multidisciplinary workforce could provide a more comprehensive understanding of the organizational dynamics within Magnet hospitals. In summary, while this study provides a foundational understanding of organizational silence and hospital magnetism, it is essential to address these limitations in future research to enhance the external validity and applicability of the findings. It should be noted that this study was conducted 5 years after the magnetic certification was completed. This time factor may have an impact on the research results. On 1 hand, hospitals have sufficient time to integrate the magnetic concept into their management systems, and the magnetic environment is relatively mature and stable. On the other hand, nurses’ perception of magnetic elements may tend to become more normalized over time, and their inhibitory effect on organizational silence may differ from that in the initial stage of certification.

## 7. Conclusions

This study is the 1st to explore the relationship between clinical nurses’ organizational silence and hospital magnetism level in the context of China’s Magnet hospitals. The results show that the organizational silence of clinical nurses in Magnet-certified hospitals is relatively low, and the hospital magnetism level is relatively high. Hospital magnetism is significantly negatively correlated with organizational silence and is an important protective influencing factor. Unmarried status, junior professional title, and poor self-perceived health are risk factors for higher organizational silence, while male gender, married status, and senior professional title are related to higher hospital magnetism perception. It is suggested that nursing managers should continuously enhance the hospital’s magnetism level by strengthening cultural value construction, optimizing the collaboration between doctors and nurses, and paying attention to the growth and psychological support of junior nurses, in order to further reduce organizational silence behavior. In the future, longitudinal studies can be conducted in hospitals at different certification stages, and multi-center studies can be carried out to better understand the long-term impact and causal relationship of Magnet hospital construction on organizational silence.

## Author contributions

**Conceptualization:** Li-Hua Shen.

**Data curation:** Hui-Ying Chen, Li-Hua Shen, Si-Jia Li, Jia Guo.

**Formal analysis:** Jia Guo.

**Funding acquisition:** Jia Guo, Wen Zhang.

**Investigation:** Si-Jia Li, Wen Zhang, Chen-Ping Zhu, Juan Tao.

**Methodology:** Li-Hua Shen.

**Project administration:** Hui-Ying Chen.

**Software:** Hui-Ying Chen.

**Supervision:** Si-Jia Li, Chen-Ping Zhu.

**Validation:** Hui-Ying Chen, Si-Jia Li, Juan Tao.

**Writing – original draft:** Si-Jia Li, Jia Guo, Wen Zhang, Chen-Ping Zhu.

**Writing – review & editing:** Hui-Ying Chen.


