# Supplementary material for: Exploring the nexus between organizational silence and hospital magnetism: A cross-sectional study on clinical nurses in China
Source: Medicine (Baltimore). 2026 Jul 31;105(31):e49928. doi: 10.1097/MD.0000000000049928 (PMC13433107; doi:10.1097/MD.0000000000049928)
Supplement: Supplementary file 1 [file medi-105-e49928-s001.pdf]

Employee Silence Behavior Survey Scale

English Version

Instructions: Please indicate the extent to which you agree or disagree with the following statements based on your feelings. "1" means strongly disagree, "5" means strongly agree, with intermediate options representing different levels of agreement. Please select the appropriate number.

Acquiescent Silence (Items 1-4)

No.	Item	Strongly Disagree 1	2	3	4	Strongly Agree 5
1	The leadership has basically made decisions, and my opinions won't make much difference.	○	○	○	○	○
2	My suggestions won't influence the current situation.	○	○	○	○	○
3	The likelihood of leadership adopting my suggestions is very small.	○	○	○	○	○
4	Leadership won't change certain decisions, so speaking up doesn't make much sense.	○	○	○	○	○

Defensive Silence (Items 5-8)

No.	Item	Strongly Disagree 1	2	3	4	Strongly Agree 5
5	To avoid affecting interpersonal relationships with colleagues, I choose to remain silent about shortcomings and oversights in others' work.	○	○	○	○	○
6	I should restrain myself and not voice my opinions to avoid becoming a target of criticism.	○	○	○	○	○
7	There's no need to offend leaders and colleagues.	○	○	○	○	○
8	I have good relationships with everyone, and for the sake of face, I'd rather not offer opinions.	○	○	○	○	○

Indifferent Silence (Items 9-12)

No.	Item	Strongly Disagree 1	2	3	4	Strongly Agree 5
9	Other people's affairs have nothing to do with me, so there's no need to comment.	○	○	○	○	○
10	I don't care about hospital affairs; it doesn't matter to me.	○	○	○	○	○
11	Adopt the doctrine of the mean; saying less means less responsibility.	○	○	○	○	○

No.	Item	Strongly Disagree<br>1	2	3	4	Strongly Agree<br>5
**12**	My emotional connection to the hospital is not deep, so there's no need to speak up.	○	○	○	○	○

Employee Silence Behavior Survey Scale - English Version

Note: This scale includes three dimensions: Acquiescent Silence (items 1-4), Defensive Silence (items 5-8), and Indifferent Silence (items 9-12).
